# Solvent and Bar Coating Parameter Selection for Optimum Deposition of Perovskite Films at Low Temperatures under Ambient Conditions

**DOI:** 10.1021/acsomega.6c06331

**Published:** 2026-09-12

**Authors:** Carlos F. Arias-Ramos, Francisco E. Cancino-Gordillo, Diana Marcela Montoya, Paola G. Abrego-Martínez, Ari M. Vargas-Sotomayor, Asiel N. Corpus-Mendoza, Hailin Hu

**Affiliations:** † Instituto de Energías Renovables, Universidad Nacional Autónoma de México. Priv. Xochicalco S/N, Temixco, Morelos 62580, México; ‡ Grupo de Películas Delgadas y Nanofotónica, Departamento de Física, Pontificia Universidad Javeriana, Cr. 7 No. 40- 37, Ed. 52 Of. 606, Bogotá 110231, Colombia; § Secretaría de Ciencia, Humanidades, Tecnología e Innovación (SECIHTI), Av. Insurgentes Sur 1582 Col. Crédito Constructor, Demarcación Territorial, Benito Juárez, Ciudad de México 03940, México

## Abstract

The transition from small-area and atmosphere-controlled spin-coating to large-area bar coating in an uncontrolled ambient process requires a comprehensive understanding of the crystallization kinetics in perovskite precursor layers. In this work, we investigate the structural, optical, and photovoltaic properties of additive-free methylammonium lead triiodide (MAPbI_3_) films fabricated via bar coating without a glove box. We analyze these properties as a function of the precursor solvent system, plate temperature, and bar groove depth. The solvent strategy employs a mixture of 2-methoxyethanol (2ME), acetonitrile (ACN), and *N*-methyl-2-pyrrolidone (NMP) to regulate nucleation and growth at low processing temperatures (24–50 °C). Our findings indicate that the volumetric ratios of the solvent components and the plate temperature are critical in determining crystallinity, optical homogeneity, and film compactness. In contrast, the bar groove depth primarily modulates film morphology and compactness, with a less pronounced effect on the material’s intrinsic structural or optical properties. By optimizing the synergy between solvent chemistry and physical coating parameters, the power conversion efficiencies (*PCE*) were enhanced from 7 to 11.85% under relative humidity >35%. This study establishes a processing protocol that may facilitate future scale-up of bar-coated perovskite films.

## Graphical abstract

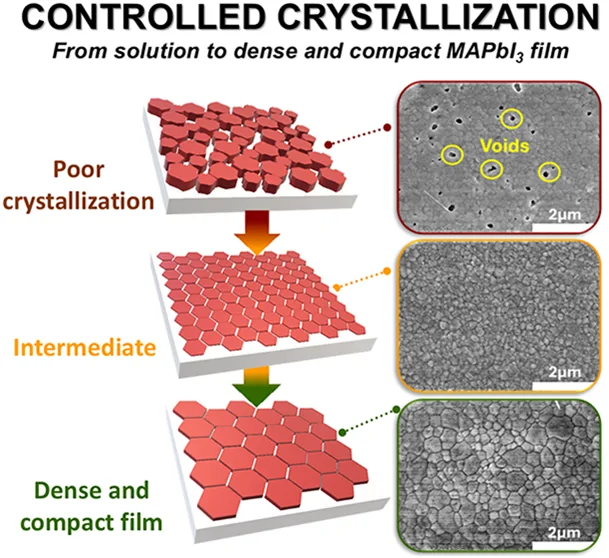

## 1 Introduction

Hybrid perovskite solar cells (PSCs) show great potential as an emerging photovoltaic technology, demonstrating electrical performance close to that of their silicon counterparts, which are the current market leaders.^1,2^ To date, the most significant advances have been achieved in small-area PSCs (< 0.1 cm^2^) fabricated in controlled atmospheres such as in an N_2_-filled glove box using laboratory-scale techniques, primarily spin-coating.^3^ While this is a practical and low-cost deposition technique, it is incompatible with industrial-scale processes^4^ due to the poor film homogeneity on substrates larger than 25 cm^2^.^5^ Another limitation is the waste of perovskite precursor solutions, which exceed 60% of the original volume due to the nature of the spinning process. Moreover, the use of an anti-solvent for crystallization control represents 70–90% of the total solvent volume in the manufacturing process, which significantly increases the production costs.^6^

To overcome these limitations, deposition techniques, such as large-area PSCs, including thermal evaporation,^7^ inkjet printing,^8^ screen printing,^9^ slot-die coating,^10^ doctor blade (DB),^11,12^ and bar coating^13^ are preferred to fabricate large-area PSCs. Among these, bar coating is a low-cost deposition technique that generates 20–30% less precursor waste per film in comparison to spin coating, and allows for uniform scaling compatible with scalable Roll-to-Roll manufacturing.^14–16^ However, challenges remain in the transition from spin-coating to bar coating, especially in perovskite compounds during the bar coating process in an uncontrolled ambient process, which is essential to obtain efficient PSCs.^17^

To obtain high-quality perovskite thin films by solution methods, it is important to achieve rapid crystal nucleation and a slow crystal growth process.^18^ For spin-coating, high-boiling-point dimethylformamide (DMF) and dimethyl sulfoxide (DMSO) are widely used as the main solvents in perovskite precursor solutions to control the crystal growth rates.^19,20^ Also, the low evaporation rates of anti-solvents have been shown to be an effective approach to introduce supersaturation and crystal nucleation in the precursor solution. However, the use of anti-solvents in bar coating remains a significant technical hurdle, because it would mean adding other elements to ensure their proper dispersion and extraction. To address these limitations, low-boiling-point solvents such as 2-Methoxyethanol (2ME) or the combination of 2ME with acetonitrile (ACN), have emerged as promising primary solvents for perovskite precursors deposited by bar coating to induce a rapid crystal nucleation.^21–23^ Even so, the quick evaporation of these solvents must be properly balanced with the coordination chemistry of the lead compounds to avoid accelerated crystal formation. For example, the inclusion of Lewis base co-solvent^24^ with high capacity of coordination (high donor numbers, *D_n_*) such as dimethyl sulfoxide (DMSO),^22,25,26^ or 2-*N*-Methylpyrrolidone (NMP)^21,27^ facilitates the formation of stable intermediate complex (NMP-MAI-PbI_2_, NMP-PbI_2_)^28^ inside the perovskite precursor solutions, providing a mechanism to reduce crystal growth rate and, at the same time, maintaining a high nucleation speed under fast-evaporating of the main solvents. Furthermore, carrying out the deposition process in uncontrolled ambient conditions such as relative humidity (RH) is a critical factor in the scalability of large-area perovskite films. Prior research on 2ME-based solvent systems for blade coating has achieved high power conversion efficiencies (*PCE*) using multi-cation/anion perovskite formulations, though typically restricted to environments with RH < 30%.^27^ Similarly, a *PCE* of 8% was reported for the standard methylammonium lead tri-iodide (MAPbI_3_) fabricated under RH < 35%.^25^ Notably, both studies rely on auxiliary drying mechanisms, such as high vacuum or infrared irradiation, prior to the final thermal annealing. While 2ME/ACN solvent systems using DMSO as a co-solvent have been explored for low-temperature bar coating,^22^ we observed that under medium-humidity conditions, DMSO exhibits limited miscibility within the 2ME/ACN system, which represents a significant challenge for film uniformity and stability in ambient processing.

In this study, we propose 2ME/ACN blend solvent systems with NMP as a coordinating co-solvent to form MAPbI_3_ perovskite film for glovebox-free processing. We systematically analyze the impact of the composition of this solvent system on the physical properties of the perovskite thin films and evaluate the relationship between this solvent system and the bar coating machine parameters, such as the temperature of the support plate and the groove depth of the coating bar. Our results demonstrate that the film thickness, compactness, and optoelectronic properties of the MAPbI_3_ layers are strictly dependent on the interplay among the solvent system and the deposition parameters. These dependencies arise from variations in nucleation and crystal growth rates driven by the specific evaporation kinetics induced by the solvent system, substrate temperature, and bar design. Ultimately, optimizing all of these parameters provides a comprehensive framework to further improve the photovoltaic performance of PSCs manufactured via scalable bar coating under uncontrolled ambient conditions.

## 2 Experimental Section

### 2.1 Perovskite Precursor Solutions

All reagents were glovebox-free processed, without further purification. Lead methylammonium triiodide (MAPbI_3_) was prepared from methylammonium iodide (MAI, 99.5%, Lumtec) and lead iodide (PbI_2_, 99.999%, Lumtec) in a 1:1 molar ratio. The precursors were dissolved in a solvent blend of 2-methoxyethanol (2ME, anhydrous, 99.8%, Sigma-Aldrich), acetonitrile (ACN, anhydrous, 99.8%, Sigma-Aldrich), and 2-*N*-methylpyrrolidone (NMP, anhydrous, 99.8%, Sigma-Aldrich) to form the precursor solutions or perovskite inks (PVK-inks). The final solution was stirred at 650 rpm without heating for 24 h prior to use.

### 2.2 Solar Cell Fabrication

The perovskite solar cells featured a configuration of glass/FTO/ETL/perovskite/HTL/Au (Figure S1). The glass with the conductive oxide layer is cut to a size of 2.5 × 2.5 cm (area = 6.25 cm^2^). The fluorine-doped tin oxide layer (FTO, TEC15, Greatcellsolar Materials) was etched with zinc powder (Purified, Fermont) and hydrochloric acid (HCl, ACS 38%, J.T. Baker) at 3 M to pattern the contacts in the final device, leaving an area with FTO of 4.75 cm^2^ (2.5 × 1.9 cm). Once etched, the substrates were sonicated in neutral detergent (pH 7.00, HYCEL), rinsed in distilled water, sonicated in acetone (ACS 99.7%, FERMONT) and isopropanol (ACS 99.9%, FERMONT), dried, and finally treated with ozone (UV Ozone Cleaner, Ossila) for 20 min.

The electron transport layer (ETL) consisted of a compact titanium dioxide (c-TiO_2_) layer and a mesoporous one (mp-TiO_2_), both processed under uncontrolled ambient conditions. The c-TiO_2_ was synthesized from a sol-gel solution consisting of 1.2 mL of titanium isopropoxide (97%, Sigma-Aldrich), 0.4 mL of hydrochloric acid (ACS 38%, J.T. BAKER), and 20 mL of isopropanol (ACS 99.9%, Fermont), sonicated for 1 h at room temperature, and then, 100 μL of the solution were deposited onto clean FTO substrates to cover an area of 4.75 cm^2^ (2.5 × 1.9 cm), at 3000 rpm, held for 30 s. Then the substrates were dried at 140 °C for 5 min, followed by a heat treatment at 450 °C for 30 min. The mp-TiO_2_ precursor solution was prepared by dissolving TiO_2_ paste (30NRD, Greatcellsolar Materials) in ethanol (ACS 99.8%, Fermont) at a 1:8 wt ratio and then stirred at 550 rpm for 5 h at room temperature. The mp-TiO_2_ solution was deposited onto the c-TiO_2_ layer by spin-coating at 4000 rpm for 30 s to cover an area of 4.75 cm^2^ (2.5 × 1.9 cm), followed by thermal annealing at 500 °C for 30 min.

On top of the ETL layers, 30 μL of PVK-ink were dispensed and dispersed by a bar using a Bar coating system (TBJ-X3-ABO, NBCHAO), shown in Figure 1a. The perovskite ink coating area is 2.5 × 1.9 cm (4.75 cm^2^), leaving 2.5 × 0.6 cm (1.5 cm^2^) FTO strip for later use. The bar coating system was placed inside a fume hood and operated under laboratory ambient conditions, without humidity control. The bars used had groove depths of 0, 4, and 12 μm (Figure 1b). During the operation, the separation distance between the bar and the nitrogen knife was 4 cm as they moved together horizontally at a speed of 20 mm/s. The plate temperature varied between 24 and 50 °C, while the N_2_ flow pressure was 25 psi, and the N_2_ knife angle was 65°.

**Figure 1:**
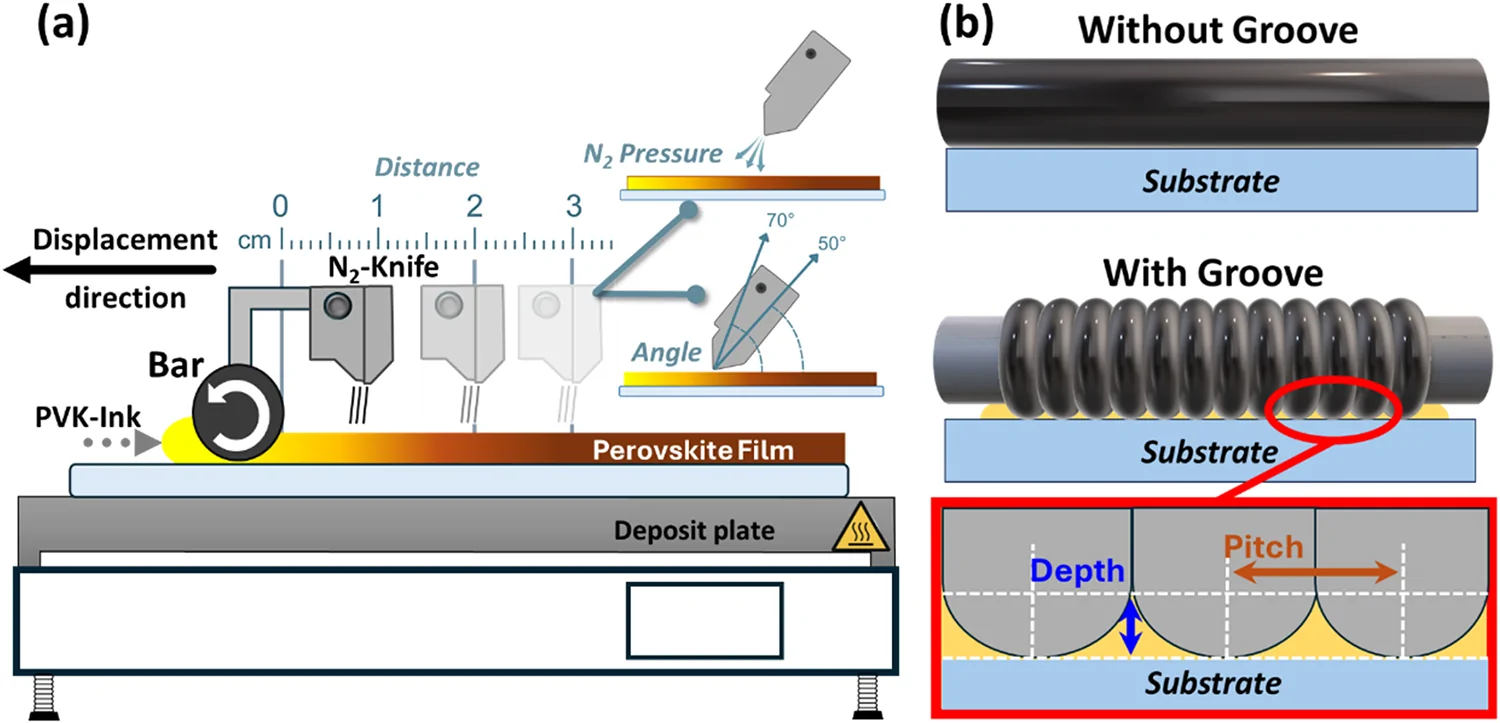
(a) Scheme of a bar coater with bar and N_2_ knife; perovskite precursor wet coating (PVK-ink) is illustrated with a yellow layer before the coating bar (black circle). (b) Bars without Groove and with different groove depth: 4 and 12 μm.

The hole transport layer (HTL) solution was prepared by dissolving Spiro-OMeTAD (99.8%, Lumtec) in chlorobenzene (ACS 99.5%, Sigma-Aldrich) at a concentration of 0.08 M, followed by adding 13.4 μL of a solution composed of 0.52 M bis­(trifluoromrthane)­sulfonimide lithium salt (99.5%, Sigma-Aldrich) in acetonitrile (ACN, anhydrous, 99.8%, Sigma-Aldrich) and 21.8 μL of 4-*tert*-butylpyridine (98%, Sigma-Aldrich). The HTL mixture was stirred for 1 h at 1000 rpm without heating, and the solution was spin-coated on perovskite films under controlled humidity (<10%) at 5000 rpm for 30 s, to cover an area of 4.75 cm^2^. Subsequently, the HTL is exposed to air for 12 h to induce oxidation.

Finally, gold metal contacts (80 nm) were thermally evaporated onto HTL (TE12, INTERCOVAMEX) at pressure of ≈2.6 × 10^–5^ Torr.

### 2.3 Material and Device Characterizations

All material and device characterizations were performed under ambient humidity. The optical absorbance (*A*), transmittance (*T*), and reflectance (*R*) of the bar-coated perovskite films were measured using a spectrophotometer (Shimadzu UV-1800) in the 300–1000 nm wavelength range at medium speed with a 2.0 nm interval. The thicknesses (*D*) and profiles of the films were obtained using a profilometer (Ambios Technology XP-200 Stylus) with a 1.5 mm scan at 0.17 mm/s and a stylus force of 5 μm.

To quantify optical dispersion of the perovskite thin films, the optical density (*OD*) spectrum of each perovskite film was integrated with respect to wavelength (λ) at 780–1000 nm region, called *OD*_int_:^29^

1 $${\mathrm{OD}}_{\mathrm{int}}=\int_{780}^{1000}A/d\left(\lambda \right)d\lambda$$

X-ray diffraction (XRD) patterns of perovskite thin films were measured on a Rigaku DMax-2200 system with a Cu–Kα radiation source at λ = 0.15406 nm, an incidence angle of 1.5°, and a diffraction rate of 2°/min. Crystal size (Φ) was estimated using the Scherrer equation:

2 $$\Phi =\frac{K\lambda }{\beta \;\cos\theta }$$

The surface morphology of thin films was analyzed by using a high-resolution scanning electron microscope (SEM) (Hitachi FE-5500) at a voltage of 5 kV.

The current density-voltage (*J*–*V*) curves of PSC’s were measured with a Keithley 2400 Sourcemeter from 1.1 to −0.1 V while the cells were illuminated under 1 sun irradiance at an air mass AM 1.5 using an Oriel LCS-100 solar simulator. The active illumination area of each cell was 0.104 cm^2^. The average and standard deviation of the electrical parameter measurements of the manufactured cells were calculated for 6 samples of each variable studied.

## 3 Results and Discussion

Figure 2 shows SEM surface images for perovskite precursor concentrations in the range from 0.4 to 1.2 M. It is observed that film coverage by bar coating improves as the concentration increases from 0.4 to 1.2 M. Similarly, XRD intensity (*I*) increases with precursor concentration due to improved compaction and increased grain size (Figure 3a). However, higher concentrations result in excessive film thickness (Figure 3b) that decreases the power conversion efficiency (*PCE*) of the solar cells (Figure 3c). On the basis of this observation, a precursor concentration of 0.8 M has been selected in this work for the rest of the study.

**Figure 2:**
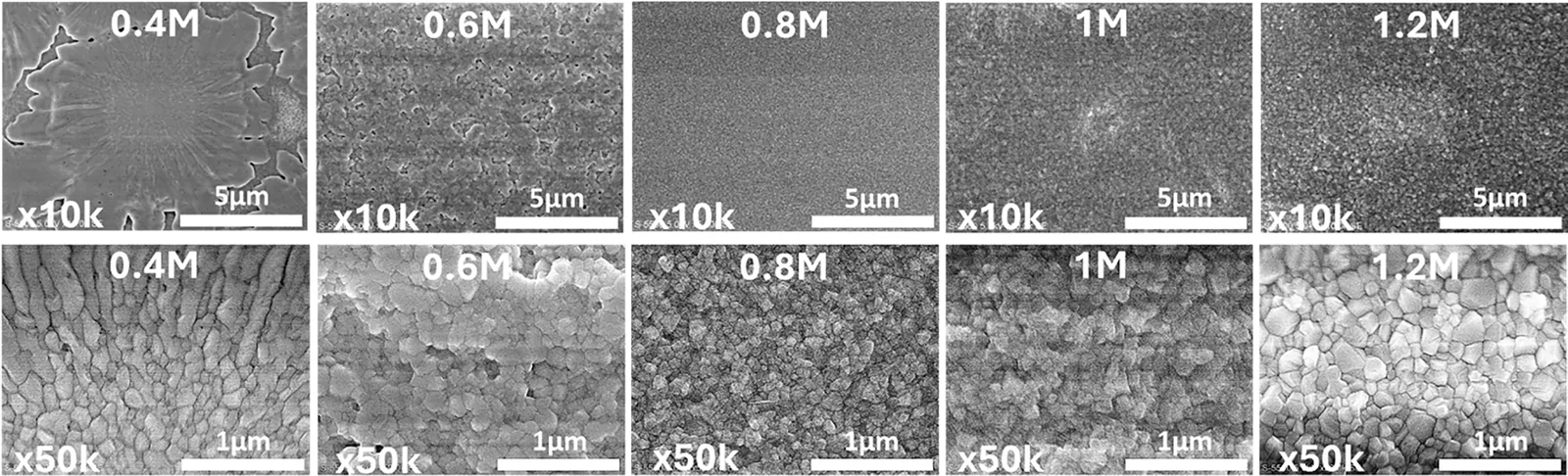
SEM surface micrographs of perovskite films prepared with different perovskite precursor concentrations (M = mol/L). Deposition conditions: temperature = 25 °C, and relative humidity = 37 %.

**Figure 3:**
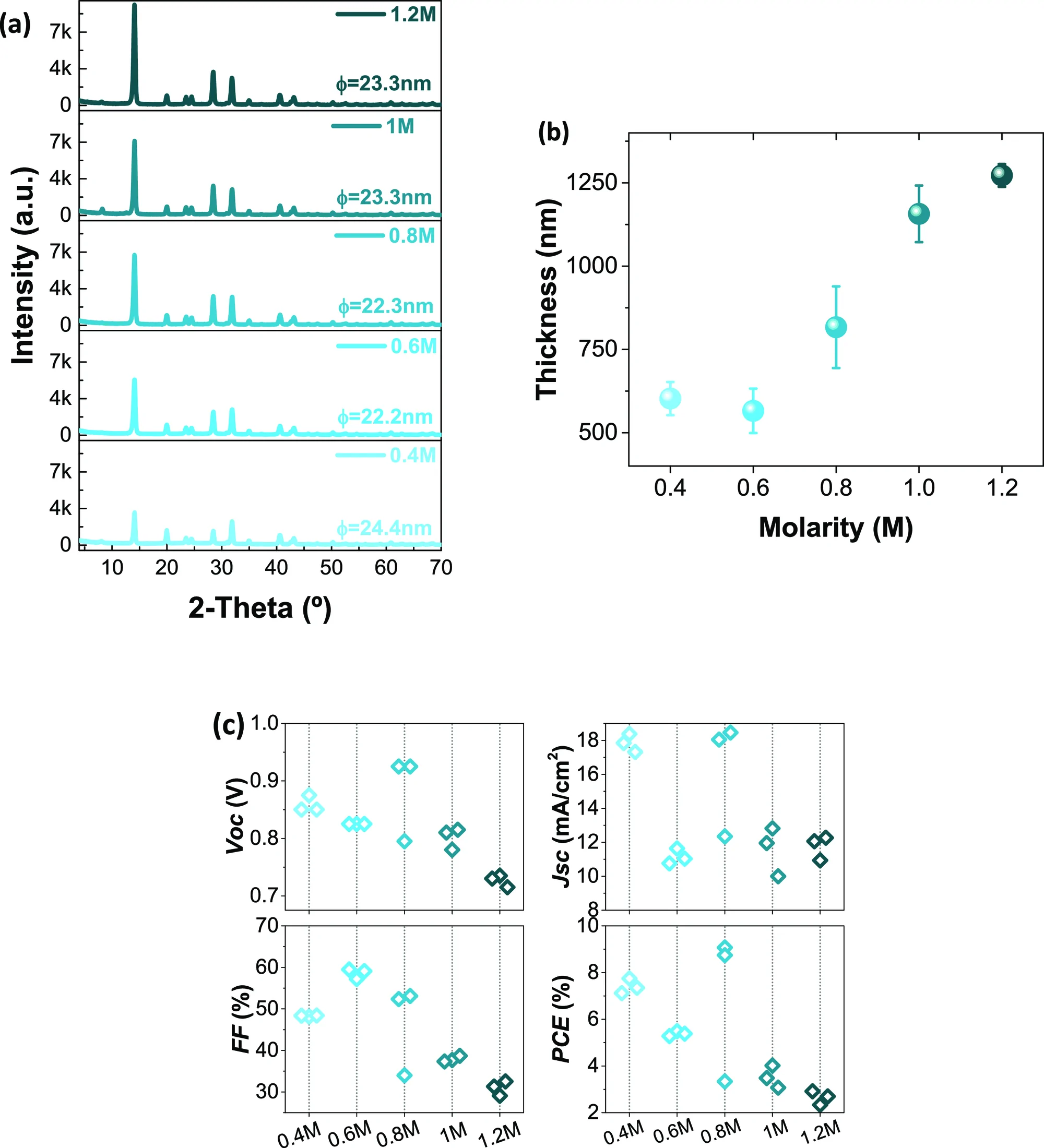
(a) XRD patterns, (b) Thickness and (c) Photovoltaic parameters (Open circuit voltage = *V_oc_*, Short-circuit current = *J*_sc_, Fill factor = *FF*, and Power conversion efficiency = *PCE*) of solar cells prepared with different concentrations of the perovskite precursor solution (ink) (M = mol/L). The solvent system was 2ME:ACN = 70:30, and NMP of 5 μL/mL. Deposition conditions: temperature = 25 °C, and relative humidity = 37%.

### 3.1 Solvent Systems for the Perovskite Precursor Solutions

The solvents were classified into three distinct categories by correlating *D_n_* with the boiling point (B.P.) and vaporization pressure (V.P.), the latter determining the evaporation rate:

(1) High-vaporization low-coordination solvents (HVLCSs) such as acetonitrile (ACN).
(2) High-vaporization medium-coordination solvent (HVMCS) such as water, 2ME, toluene, and chlorobenzene (CB).
(3) Low-vaporization high-coordination solvents (LVHCSs) include dimethylformamide (DMF), dimethyl-sulfoxide (DMSO), *N*-methyl-2-pyrrolidone (NMP), and γ-butyrolactone (GBL).

Table 1 summarizes the *D*_*n*_, B.P., and V.P. values for ACN, 2ME, and NMP, compared to DMF and DMSO, the benchmark solvents typically utilized in spin-coating processes. Building on these properties, the solvent systems developed for the perovskite precursor in this work are based on a dual-component strategy: (*i)* a high-volatility solvent matrix, consisting of a 2ME:ACN volumetric mixture (% v/v), designed to promote rapid precursor supersaturation and crystal nucleation at low substrate temperatures, and (*ii)* a high-coordination, low-evaporation solvent, specifically NMP, which acts as a retardant to ensure controlled crystal growth and the formation of a high-quality intermediate phase.

**Table 1:** Characteristics of Different Solvents for Perovskite Precursor Solutions

**Type of solvent**	**Solvent**	**Donor number (*D*** *_**n**_*, kcal/mol)	**Boiling point** (B.**P**., **°C)**	**Vaporization pressure** (V.**P**., **Pa at 20 °C)**
HVLCS	Acetonitrile (ACN)	14.1	82	9700
HVMCS	2-Methoxylethanol (2ME)	20.0	124	800
LVHCS	*N*-Methyl-2-pyrrolidone (NMP)	27.2	202	39
LVHCS	Dimethyl-formamide (DMF)	26.6	153	350
LVHCS	Dimethyl-sulfoxide (DMSO)	29.9	189	55.9

#### 3.1.1 Effect of Rapid Vaporization Solvents: The Combination of 2ME and ACN

The low B.P. of 124 °C for 2ME makes it an unsuitable candidate to induce spontaneous evaporation during low-temperature deposition, in comparison to DMF with a higher B.P. of 153 °C. However, mixing 2ME with ACN, B.P. (82 °C), facilitates solvent evaporation at lower temperature. By selecting different proportion between 2ME and ACN, three scenarios can be established: (1) high solvent evaporation rate (HER) with a higher ACN content (2ME:ACN = 50:50); (2) medium solvent evaporation rate (MER) with an intermediate ACN content (2ME:ACN = 70:30); and (3) low solvent evaporation rate (LER) with a low ACN content (2ME:ACN = 90:10). A higher percentage of ACN is expected to induce a faster evaporation rate, accelerating the nucleation and crystal growth processes, as illustrated in Figure 4a,b. Indeed, the variation in evaporation rate has a direct impact on the morphology of the perovskite films prepared by bar coating in air, as shown in SEM micrographs in Figure 4c. In the HER case, a perovskite film is formed with grains with an average size of 260.4 nm (±101.4 nm) in which voids are observed due to rapid crystal growth that impedes grain compaction, a result of the high vaporization rate caused by the large volumetric fraction of ACN in the solvent system. Conversely, MER or LER scenarios slow down the solvent evaporation speed, allowing for better-controlled crystal nucleation and growth processes, leading to a more compact perovskite thin film with average grain size of 195.10 (±79.26) and 378.45 (±159.5) nm, respectively.

**Figure 4:**
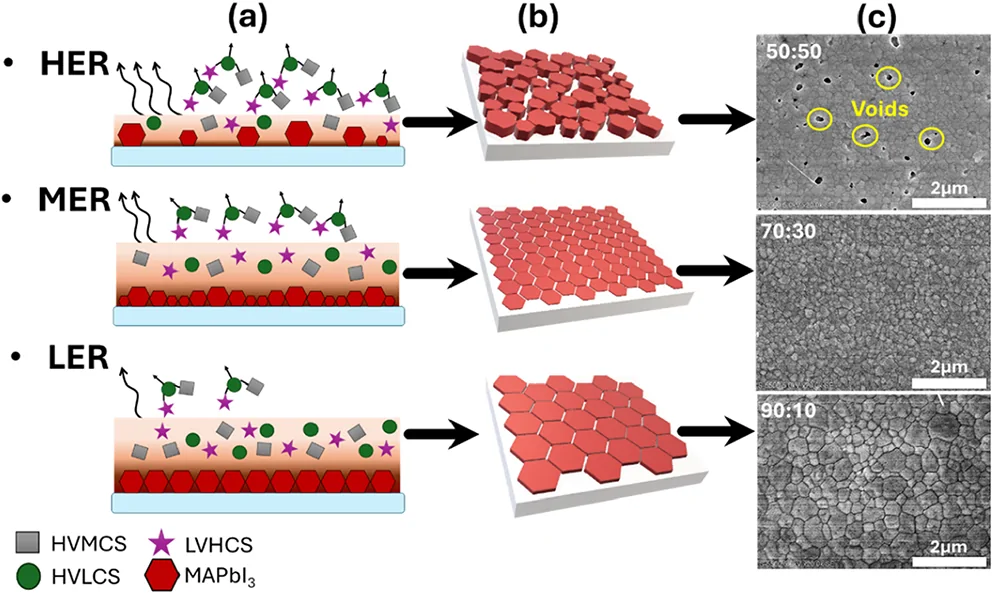
(a) Diagrams of perovskite film formation from high (HER), medium (MER), and low (LER) vaporization rate solvent systems using HVMCS (2ME), HVLCS (ACN), and LVHCS (NMP) solvent systems that lead to (b) different surface morphologies of perovskite films. (c) SEM images of perovskite films prepared from high (HER, 2ME:ACN = 50:50), medium (MER, 2ME:ACN = 70:30) and low (LER, 2ME:ACN = 90:10) vaporization rate solvent systems. Deposition conditions: temperature = 23.9 °C, and relative humidity = 39%.

The impact of the evaporation rate on the crystallinity and optical properties of perovskite films was investigated as a function of three 2ME:ACN volumetric ratios (50:50, 70:30 and 90:10). XRD patterns for all samples exhibit the characteristic tetragonal phase of MAPbI_3_, with the main peak at approximately 14.1°, corresponding to the (110) lattice plane (Figure 5a).^30^ Among the tested conditions, the films with the highest ACN content (50:50) show the lowest diffraction intensity, the smallest crystal size (Φ), and a crystalline impurity at approximately 8°, which corresponds to an intermediate PbI_2_–NMP adduct.^31^ On the other hand, the optical density (*OD*) spectra (Figure 5b) confirm a well-defined optical absorption edge at around 780 nm for all samples, consistent with literature reports for MAPbI_3_.^32^ Notably, higher *OD* values were observed in the 580 – 780 nm range for films prepared with increased ACN concentration (50:50, 60:40 and 70:30). Since 780 nm corresponds to the optical bandgap of these perovskite films, photons with longer wavelength should theoretically not be absorbed. However, the presence of a non-zero baseline in the near-infrared region (780–1000 nm) suggests increased optical losses associated with film inhomogeneity (inset in Figure 5b). These losses may originate from several contributions, including light scattering, thickness fluctuations, surface roughness, voids, and structural defects.^33,34^ To further support this interpretation, complementary reflectance and transmittance spectra are provided in the Supporting Information (Figures S2–S5), which show trends consistent with the optical density analysis and the corresponding SEM, profilometry, XRD, and photovoltaic measurements. Therefore, the OD baseline is interpreted as a complementary descriptor of the overall optical quality of the films rather than as a direct measurement of a single morphological parameter.

**Figure 5:**
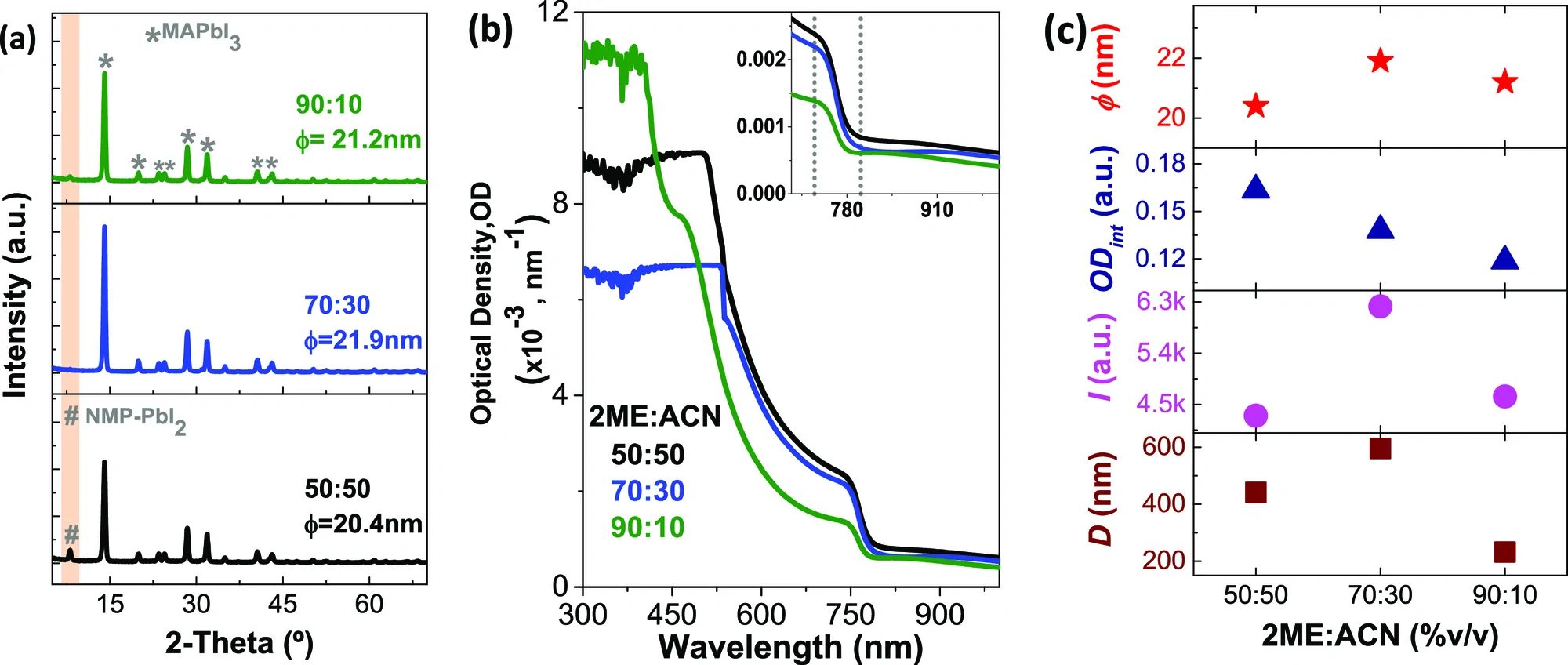
(a) XRD patterns and (b) optical density (*OD*) of three perovskite thin films prepared with % v/v of 2ME:ACN = 50:50, 70:30 and 90:10. (c) Film thickness (*D*), intensity of the XRD peak at 14.1° (*I*), integrated optical density at 780–1000 nm region (*OD*_int_) and average crystal size (Φ) of the three perovskite film samples. Inset graph in (b) is a zoom of the optical density curves in a range of 700 to 1000 nm, with emphasis on the absorption edge of the perovskite film. Deposition conditions: temperature = 23.9 °C, and relative humidity = 39%.

To quantify this effect, the *OD* spectra were integrated (*OD*_int_) within the 780–1000 nm range. The resulting values are plotted in Figure 5c alongside the film thickness (*D*), Φ, and the (110) *I*. The elevated *OD* *_int_* values for the 50:50 and 90:10 ratios suggest higher optical scattering, which correlates with lower XRD intensities and reduced crystallite sizes. While a well-controlled crystallization process typically yields a linear correlation between film thickness and XRD peak intensity,^35^ ,^36^ this relationship deviates significantly at high ACN concentrations (50:50). This suggests that the rapid vaporization induced by ACN triggers and accelerates stochastic crystal growth. Such a regime leads to structurally, morphologically, and optically disordered perovskite films, whereas intermediate 2ME:ACN ratios promote a more regulated growth mechanism as can be seen in the SEM images in Figure 4c.

#### 3.1.2 Effect of High-Coordination Solvent: the Use of NMP

While DMSO is the most frequently reported LVHCS for bar coating,^37^ we observed that its concentration within 2ME:ACN-based precursor solutions is strictly limited under laboratory ambient conditions. Figure 6a shows images of four MAPbI_3_ precursor inks (2ME:ACN = 70:30) containing 10–13 μL/mL of DMSO. Although initially transparent and yellow, solutions with 11–13 μL/mL of DMSO began to solidify after 720 min, rendering them unsuitable for coating. In contrast, NMP-based inks (Figure 6b) remained stable beyond 720 min, even at significantly higher concentrations (up to 40 μL/mL). The precipitation observed with DMSO is attributed to its higher donor number (*D*_*n*_ = 29.9 kcal/mol) compared to NMP (*D*_*n*_ = 27.2 kcal/mol). The stronger coordination of DMSO with the Pb^2+^ precursors promotes the formation of (DMSO)_2_MA_2_Pb_3_I_8_ intermediate phase, which exhibits limited solubility in 2ME.^26^ Consequently, for the 2ME:ACN solvent system, the use of NMP instead of DMSO allows a larger volume of LVHCS to better control the perovskite crystallization kinetics. Figure 6c shows the surface morphology of the perovskite films obtained with 0, 20, and 40 μL/mL of NMP in a 2ME:ACN (70:30 % v/v) solvent system. In the absence of NMP, rapid crystallization yields grains with an average diameter of 211 ± 82.8 nm. Adding 20 μL/mL of NMP initially reduces the grain size, (102 ± 39.15 nm) and introduces surface artifacts, such as fibers and flakes, likely due to an inhomogeneous NMP distribution. However, increasing the NMP volume to 40 μL/mL results in compact morphology with larger grain (306 nm ± 117), consistent with the role an LVHCS in regulating crystal growth.

**Figure 6:**
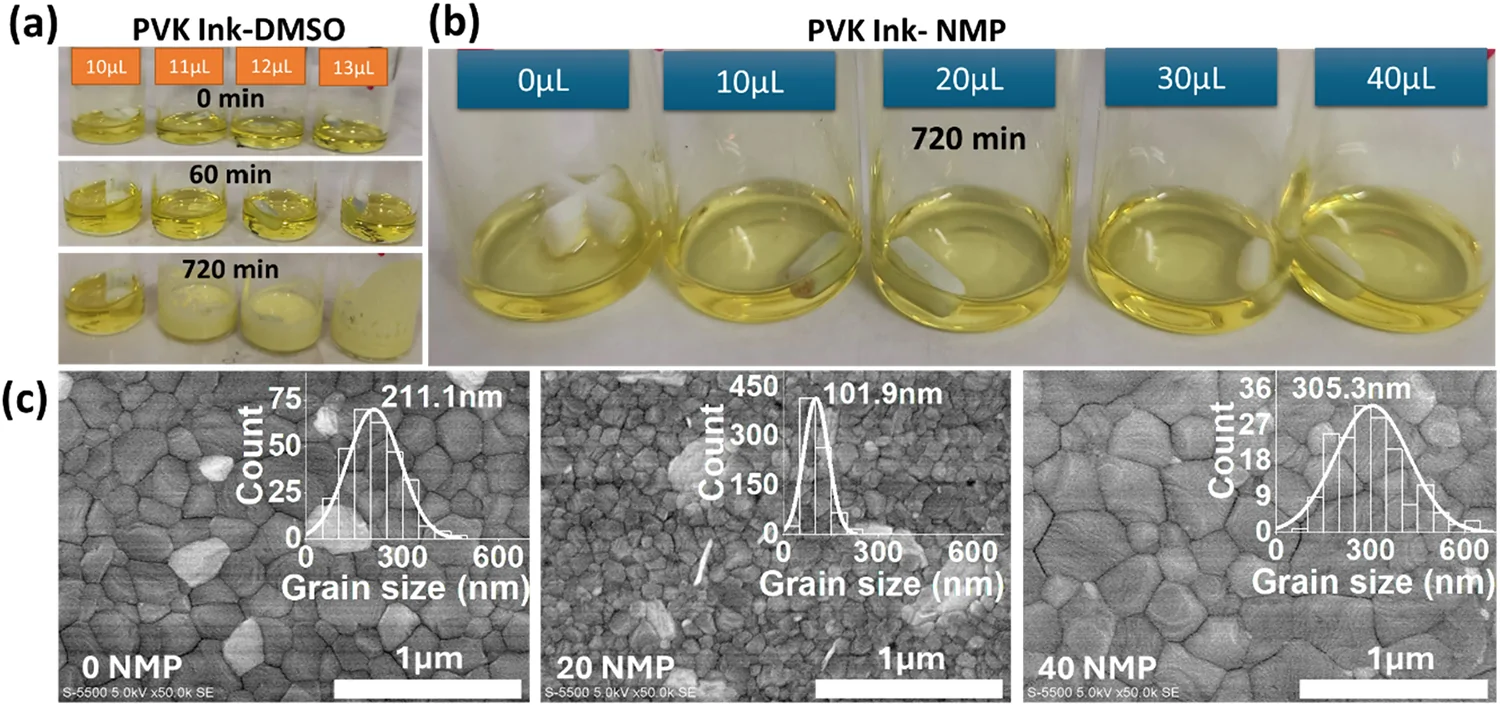
Photographs of 2ME:ACN-based perovskite precursor solutions (PVK ink) with different volume concentration of (a) dimethyl sulfoxide (DMSO): 10 to 13 μL/mL, at 0, 60, and 720 min, and (b) *N*-Methyl-2-Pyrrolidone (NMP): 0 to 40 μL/mL after 720 min. (c) Surface SEM micrographs of perovskite films fabricated by bar coating using 0, 20, and 40 μL/mL of NMP in 2ME:ACN-based perovskite precursor solutions. Deposition conditions: temperature = 23.8 °C, and relative humidity = 37%.

To further elucidate these effects, Figure 7a correlates *D*, *I*, Φ, and *OD*_int_ values across varying NMP concentrations (0–40 μL/mL). Low NMP content results in thicker films (550–700 nm), higher optical scattering, and smaller crystallites due to rapid crystallization. A critical transition occurs at approximately 20 μL/mL of NMP; beyond this point, further additions reduce both film thickness and optical scattering while significantly enhancing crystallinity. This confirms that a higher fraction (30 or 40 μL/mL) of the high B.P. and *D*_*n*_ solvent like NMP slows the crystallization rate, promoting structurally superior and more compact thin films.

**Figure 7:**
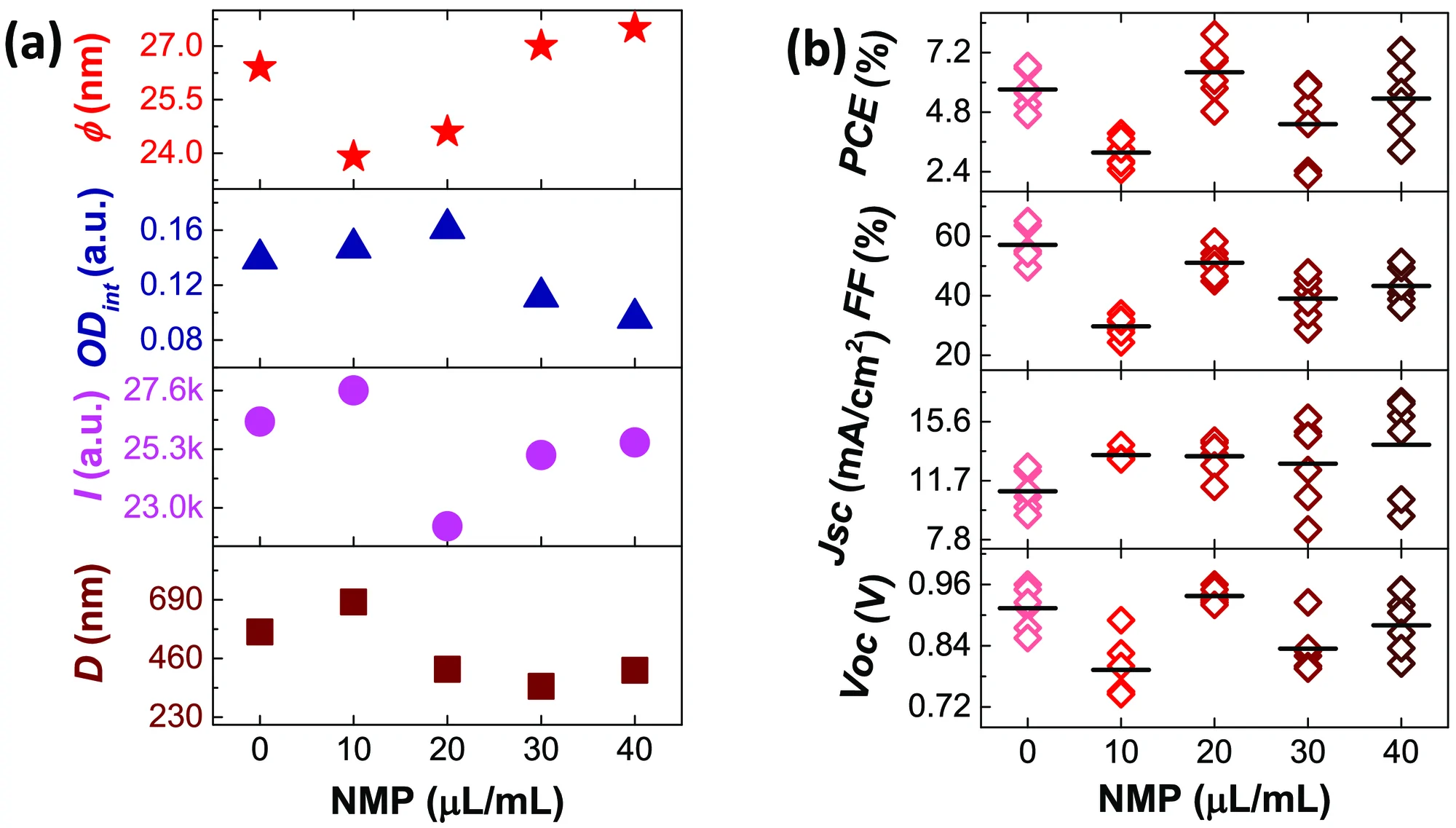
(a) Film thickness (*D*), intensity of the XRD peak at 14.1° (*I*), integrated optical density at 780–1000 nm region (*OD*_*int*_) and average crystal size (ϕ) of the perovskite film sample prepared with volume concentrations of NMP as 0, 10, 20, 30, and 40 μL/mL (2ME:ACN = 70:30 % v/v). (b) Photovoltaic parameters of perovskite solar cells with the same perovskite thin films. Deposition conditions: temperature = 23.8 °C, and relative humidity = 37%.

Figure 7b shows the photovoltaic parameters of the resulting devices. The open-circuit voltage (*V_OC_*) and the fill factor (*FF*) values show a nonlinear trend, dictating the overall *PCE*. The initial drop in *V_OC_* and *FF* at low NMP concentration is likely due to poor film quality and high optical dispersion, as shown in Figures 6c and 7a. This is a product of the null or low presence of the strong, low vaporization coordinating solvent such as NMP. The formation of large grains is promoted but with a high rate of imperfections along the surface for the concentration of 0 μL/mg of NMP. Or where the limited presence of NMP (10 ul/mg) promotes the deceleration of the crystallization process, promoting the reduction of the rate of imperfections on the film surface. A similar effect was presented by Chiu et al. in the year 2025,^26^ where the presence of 0 and 20 mol % of NMP decreases the crystallization and compaction of the perovskite films formed.

In contrast, the electrical performance shows a modest enhancement for NMP concentrations exceeding 20 μL/mL, likely resulting from improved crystallinity and lower optical scattering losses. Nevertheless, the presence of the NMP–PbI_2_ impurity phase may limit the extent of these improvements. Notably, the *J*_SC_ shows a consistent increase with NMP concentration of the synergistic effect of enhanced crystalline, reduced film thickness (facilitating charge transport), and minimized optical scattering up to 40 μL/mL. Despite these structural improvements, excess NMP may leave residual NMP-PbI_2_ impurities on the film surface (Figure S1), potentially impacting photovoltaic performance such as the *FF* which decreases as the proportion of NMP increases.^26,28,38^

### 3.2 Parameters of the Bar Coating System

#### 3.2.1 Plate Temperature

The primary effect of the plate temperature is evident in the film morphology (Figure 8a), where the average grain size increases as the plate temperature increases from 24 to 40 °C. However, upon reaching 50 °C, significant morphological defects and large surface voids appear. This structural degradation is accompanied by a simultaneous decrease in *D*, *I*, and Φ, while *OD_int_* increases (Figure 8b).

**Figure 8:**
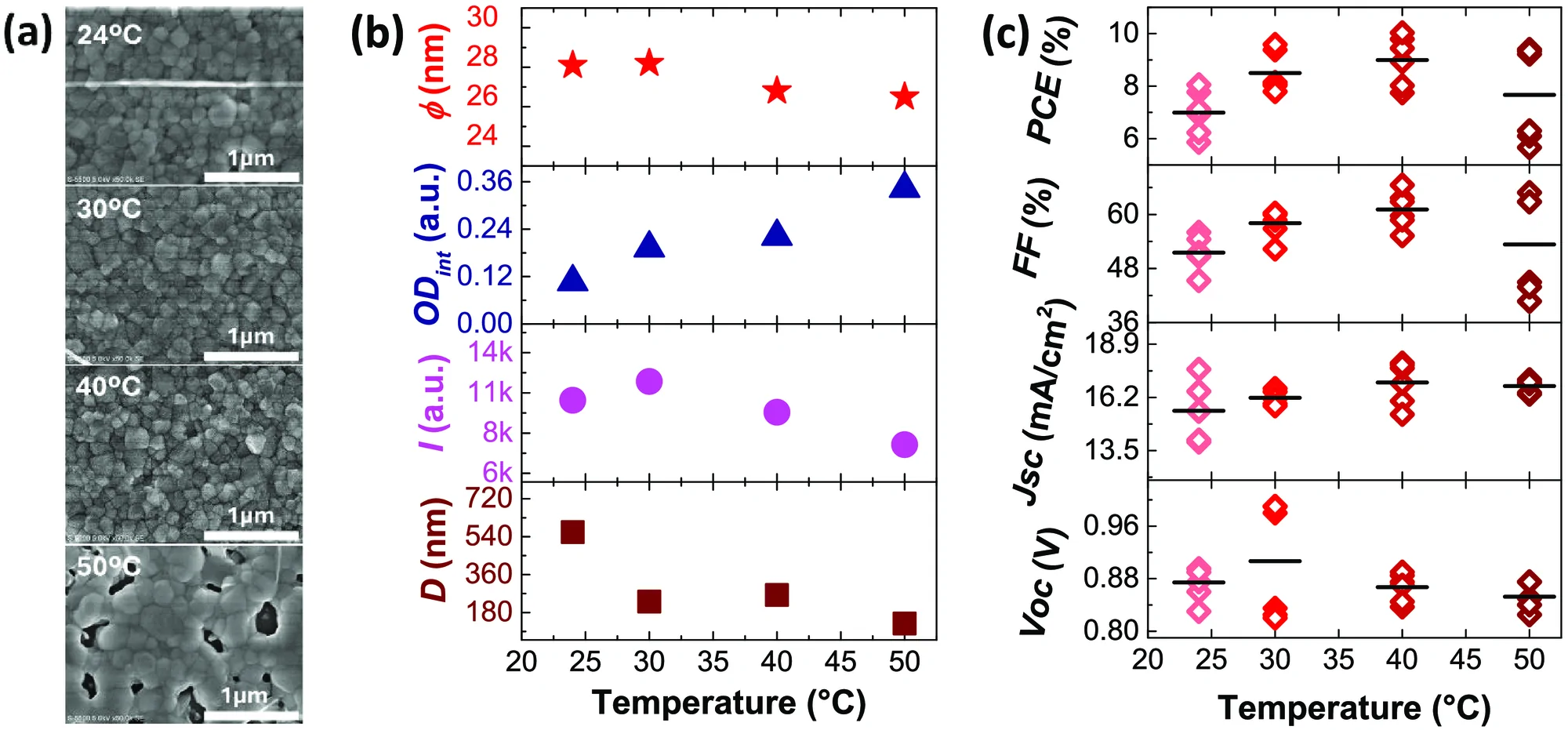
(a) SEM micrographs, (b) film thickness (*D*), intensity of the XRD peak at 14.1° (*I*), integrated optical density at 780–1000 nm region (*OD*_int_), and average crystal size (Φ) of the perovskite film sample prepared at plate temperatures of 24, 30, 40, and 50 °C. (c) Photovoltaic parameters of the solar cells with the same perovskite films. Deposition conditions: temperature = 23.4 °C, and relative humidity = 34%.

These results suggest that for the 2ME:ACN + NMP system (70:30 + 40) under ambient conditions, the support plate temperature should be maintained at or below 40 °C to ensure regulated crystal growth process. The influence of the plate temperature extends beyond simply accelerating solvent evaporation. During bar coating, crystallization is governed by the dynamic balance between solvent removal, precursor supersaturation, nucleation density, and crystal growth.^22^ Increasing the substrate temperature increases the evaporation rate of the volatile ACN component while only moderately affecting the less volatile 2ME and NMP solvents. Consequently, supersaturation is reached more rapidly, promoting earlier nucleation events. At moderate temperatures (30–40 °C), this faster solvent removal shortens the residence time of the wet film without completely disrupting the solvent-coordination environment provided by NMP. Under these conditions, the intermediate coordination complexes remain sufficiently stable to sustain controlled crystal growth after nucleation, allowing neighboring nuclei to merge into larger crystallites and producing compact films with reduced structural disorder.

However, when the plate temperature reaches 50 °C, the evaporation kinetics become excessively fast. The precursor solution rapidly loses ACN before an adequate redistribution of the remaining solvent mixture can occur, leading to a steep supersaturation gradient across the wet film. This condition increases the instantaneous nucleation rate while simultaneously shortening the time available for grain coalescence and defect healing. As a result, crystal growth becomes transport-limited rather than thermodynamically controlled, generating isolated grains separated by voids and pinholes, as observed in the SEM images. This interpretation is consistent with the simultaneous decrease in the XRD peak intensity and crystallite size, together with the increase in *OD_int_*, indicating greater optical scattering caused by morphological inhomogeneity. These observations suggest that the plate temperature defines an optimum processing window in which nucleation and crystal growth remain balanced.

Below this optimum, solvent removal is relatively slow, delaying crystallization and limiting grain development. Above the optimum, solvent evaporation dominates the process, causing premature solidification before complete crystal coalescence can occur. Therefore, a plate temperature of approximately 40 °C provides sufficient thermal energy to accelerate processing while preserving the coordination-mediated crystallization mechanism established by the 2ME:ACN:NMP solvent system.

Finally, the photovoltaic parameters (Figure 8c) mirror the trends observed in material properties. Slightly higher *V_OC_* values are obtained for samples prepared at lower temperatures (24 and 30 °C), while the *FF* improves with temperatures up to 40 °C, followed by a sharp decline at 50 °C. The above results in a better alignment of the energy levels of the perovskite and optimized interfaces with the device transport layers, because of a better formed film as a result of a lower degree of supersaturation in the precursor solution.^39^ Conversely, the *J*_SC_ remains relatively stable across all conditions. Consequently, the *PCE* follows the trend of the *FF*, with the peak performance achieved at 40 °C due to superior film compactness. Beyond this optimal temperature, the formation of surface voids leads to a significant reduction in device efficiency. From this, we can infer that increasing the plate temperature not only accelerates solvent evaporation but also modulates crystal nucleation and growth by providing additional thermal energy to the precursor film.

#### 3.2.2 Bar Groove Depth

During the bar coating process, a precursor solution meniscus forms at the leading edge of the bar.^15,22^ As the bar advances in direct contact with the substrate, the grooves penetrate the liquid, depositing a wet perovskite film in their wake.^13,15,36,40^ Figure 9a illustrates the formation of these wet coatings as a function of the groove depth. The volume and effective surface area of the wet film are highly dependent on the groove depth.^41^ SEM micrographs (Figure 9b) reveal that films deposited using the 12 μm grooved depth bar exhibit a more compact and homogeneous surface morphology than those prepared with 0 and 4 μm grooves. Although the average grain size does not increase monotonically with groove depth, the 12 μm condition produces a narrower grain-size distribution and fewer intergranular voids, indicating a more controlled crystallization process. Profilometry measurements confirm that the groove depth significantly influences film thickness; bars with 4 and 12 μm grooves produce thinner films than the 0 μm groove bar (Figure 9c). Furthermore, the increase in *I* and Φ (Figure 9b) suggests that grooved bars effectively enhance the relative crystallinity of the films (Figure 9c). A deeper groove deposits a larger volume of precursor solution, which increases the wet-film thickness and prolongs solvent evaporation time. This extended drying period promotes a more homogeneous nucleation and crystal-growth process, allowing neighboring grains to coalesce more efficiently and producing a compact film with reduced void density. Consequently, the improvement is primarily associated with grain-size uniformity and reduction of pinholes rather than with a simple increase in the average grain size. While the 4 μm bar produced the largest crystallite size, it also resulted in the highest optical scattering. In contrast, the 12 μm bar yielded the optimal balance of low optical dispersion, because of minor voids and high crystallinity, leading to the best overall photovoltaic performance (Figure 9d). The photovoltaic parameters (*J_SC_*, *V_OC_*, *FF*, and *PCE*) show a linear correlation with groove depth, confirming that the synergy between morphology, less pinholes, and optical quality is the origin for high *PCE* values. This understanding shows that these qualities determine how photoinduced charges are generated, transported, and recombined within the solar cell. Therefore, we can settle that a deeper groove increases the exposed surface area of the precursor solution. Consequently, a larger groove depth facilitates a broader evaporation front, promoting higher nucleation density and the formation of a more compact film.

**Figure 9:**
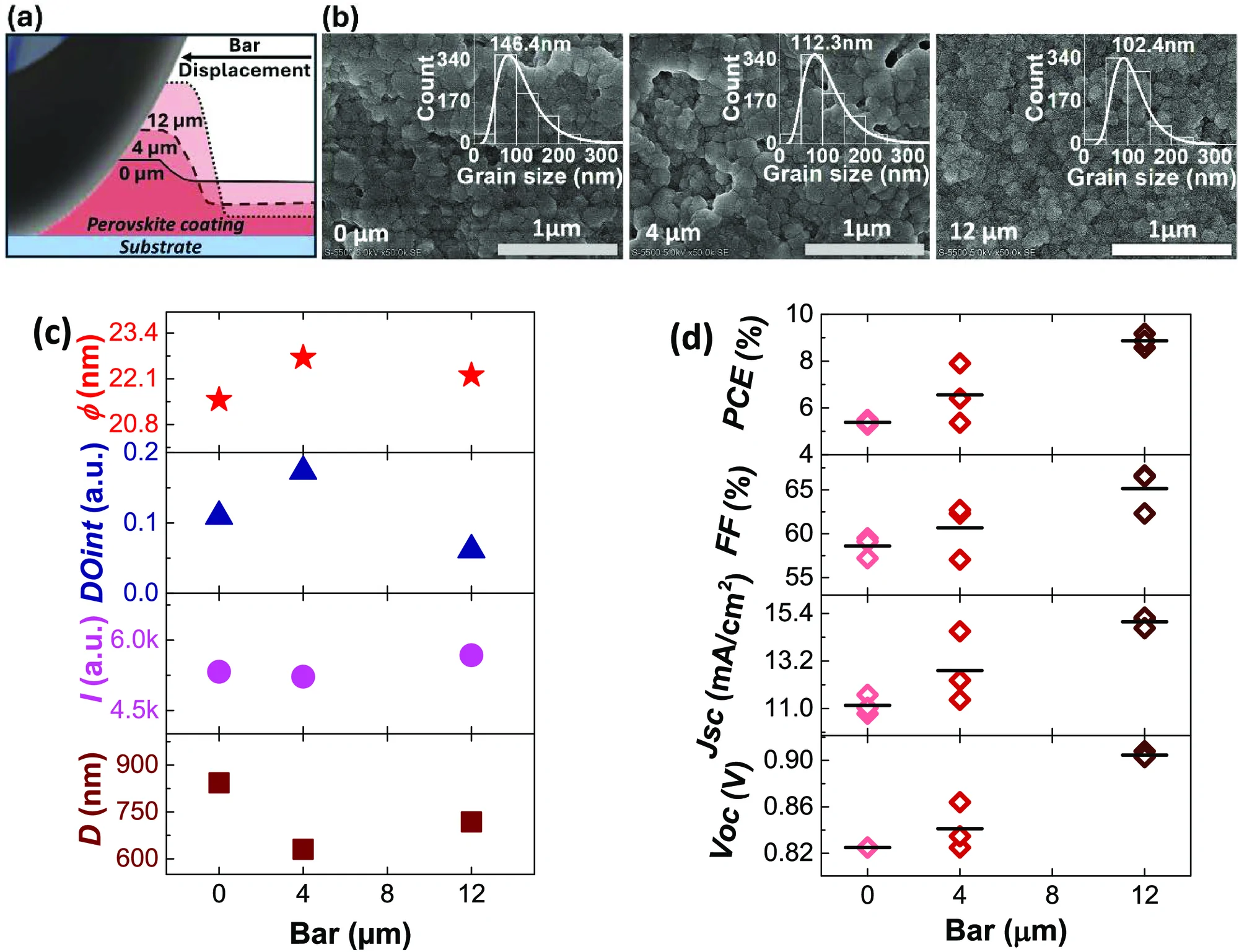
(a) Scheme of wet perovskite film after the coating bar passing through; the bar groove depths: 0, 4, and 12 μm. (b) SEM micrographs of perovskite thin films deposited by those bars. (c) Film thickness (*D*), intensity of the XRD peak at 14.1° (*I*), integrated optical density in the 780–1000 nm region (*OD*_*int*_), and average crystal size (Φ) of the three types of perovskite thin films; and (d) photovoltaic parameters of perovskite solar cells with the same perovskite films. Deposition conditions: temperature = 23.8 °C, and relative humidity = 37%.

### 3.3 Deposition Parameters Evaluation

The results presented in this work demonstrate that the interplay between the solvent system chemistry and the physical bar coating parameters fundamentally dictates the quality of perovskite films deposited under uncontrolled ambient conditions. As shown in the heatmap in Table 2, the colors range from lightest to darkest blue, representing the degree of optimization, from least to most optimized condition for film enhancement and overall quality, according to each category:

- Crystallinity: This was calculated using the crystal size of the main peak in the XRD spectra corresponding to 14.1°. The optimized film (dark blue) has the largest crystal size.
- Optical homogeneity: This was calculated using the *OD*_*Int*_ value obtained from the absorbance spectra. The optimized film (dark blue) has the lowest *OD*_*int*_ value.
- Surface Morphology: This was calculated from the surface roughness (*R*_a_), obtained from the surface profile of the films, as described in the Supporting Information (eq S1). The optimized film (dark blue) has the lowest *R*_a_ value.
- Grain Size Deviation: This was calculated using the standard deviation of the grain size obtained from the SEM micrographs of each variable. The optimized one (dark blue) has the lowest standard deviation value.

**Table 2:**
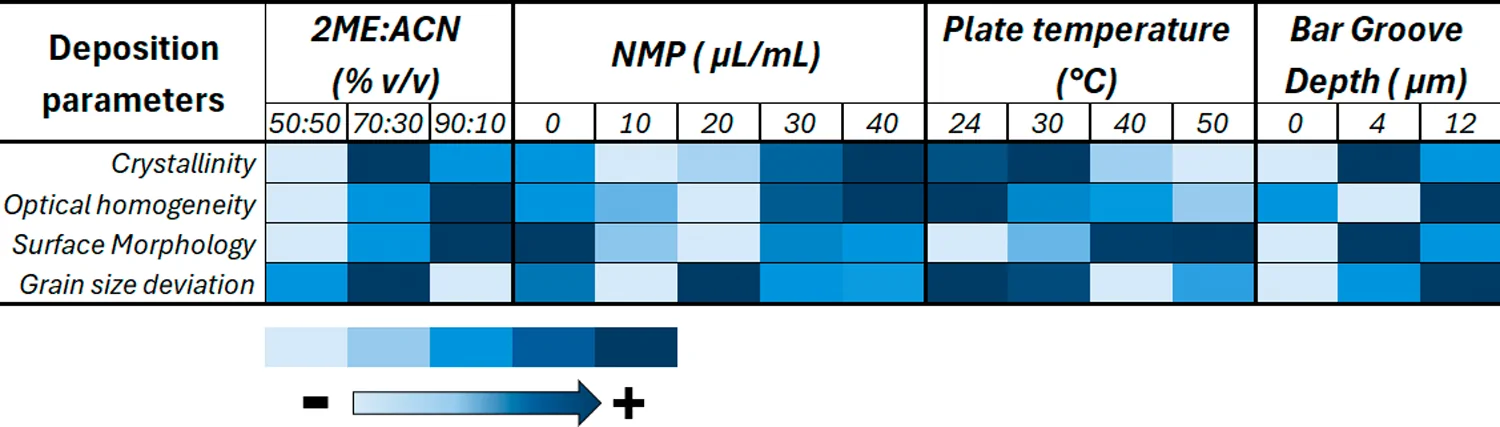
Summary of Physical Properties of Perovskite Thin Films as Functions of Deposition Parameters by Bar Coating in Air

Our experimental matrix identifies the 2ME:ACN solvent ratio and the NMP coordination co-solvent as the primary drivers of crystal formation and optical homogeneity. A 2ME:ACN ratio of 70:30 promotes superior crystallinity, optical homogeneity, and minor grain size deviation, whereas increasing the ACN content (50:50) drastically degrades film quality, leading to poor optical uniformity and reduced efficiency. Furthermore, NMP acts as a crucial retarding agent; a volume of 30 to 40 μL induces a balanced crystallization rate. In contrast, lower NMP volumes result in rapid, disordered crystal growth that compromises surface morphology.

Regarding the bar coating system, both plate temperature and groove depth significantly impact the physical properties of the perovskite films. Increasing the plate temperature from 24 to 40 °C provides the necessary thermal energy to achieve compact films and higher device performance. However, exceeding this threshold (at 50 °C) accelerates crystal growth excessively, increasing optical scattering and decreasing crystal growth, due to the increase in holes and other imperfections on the surface. Simultaneously, a groove depth of 12 μm appears to favor surface morphology, likely by optimizing the drying kinetics.

Based on these correlations, a methodology for perovskite bar coating under uncontrolled ambient processing was established: a 2ME:ACN system (70:30) precursor solution with 40 μL/mL of NMP, a plate temperature of 40 °C, and a bar groove depth of 12 μm. Under these conditions, the current density-voltage (*J*–*V*) characteristics (Table 3) yielded a maximum PCE of 11.85%, with a *V*_*OC*_ = 0.92 V, *J*_*SC*_ = 18.87 mA/cm^2^, and an *FF* = 69.79%. Using the simple MAPbI_3_ without additives, this work demonstrates that precise control over solvent systems and deposition parameters enables the fabrication of high-quality perovskite films processed glovebox-free. Defining an additive as a chemical compound intentionally incorporated in small quantities to improve, modify, or protect the primary properties of the material,^24^ in this case the perovskite film.

**Table 3:** Electrical Parameters (Average ± Deviation (Maximum) of Six Samples) of Perovskite-MAPbI_3_ Solar Cells Fabricated by Bar Coating Glovebox-Free Processing, Using 2ME:ACN (% v/v 70:30), 40 μL/mL NMP, 12 μm of Groove Depth in the Bar^a^

**Electrical Parameter**	**Average** *±* **Standard Deviation**	**Maximum Value**
***V***_**OC**_ **(V)**	0.86 ± 0.06	(0.92)
***J***_**SC**_ **(mA/cm** ^**2**^ **)**	17.85 ± 1.35	(18.87)
***FF* (%)**	66.08 ± 4.78	(69.78)
***PCE* (%)**	10.18 ± 1.86	(11.85)

a The substrate temperature: 30 °C. Deposition conditions: temperature = 25.6 °C, and relative humidity = 38%.

An important aspect of the proposed methodology is that all films were deposited under uncontrolled laboratory ambient conditions (34–39% RH), without the use of humidity-control systems. Although this humidity range is moderate, ambient fluctuations in moisture still influence precursor coordination and crystallization kinetics. Therefore, demonstrating reproducible film formation under these conditions provides a practical processing framework that is compatible with scalable manufacturing environments. Based on previous reports,^28,31,41,42^ we identify two primary sources of water in our process: (i) residual water present in the precursor solution, originating from the reagents, and (ii) moisture from the surrounding atmosphere during the crystallization phase. Water can act as a coordinating ligand in the precursor solution, forming complexes such as PbI_2_(H_2_O)_*x*_ and PbI_2_(NMP)_*x*_(H_2_O)_*y*_.^43^ This coordination arises from the lone electron pairs on the oxygen atom, which readily interact with Pb^2+^ ions.^43^ During film formation, particularly in precursor systems containing DMF, DMSO, or NMP, water competes with these solvents for lead coordination sites, promoting the formation of intermediate crystallization phases.^43^

Controlled amounts of moisture have been reported to be beneficial during perovskite film formation.^44^ Moderate water incorporation slightly retards nucleation, promoting the growth of larger and more uniform grains, reducing defect density, and increasing charge-carrier lifetime, ultimately leading to improved photovoltaic performance. However, at relative humidity levels of approximately 30–80%, the likelihood of forming hydrated degradation products, such as CH_3_NH_3_PbI_3_·H_2_O and (CH_3_NH_3_)_4_PbI_6_·2H_2_O, increases significantly, compromising the long-term stability of the perovskite films.^43,45^ Consequently, this factor may limit the processing window and manufacturing scalability of the proposed methodology.

In addition to regulating crystallization kinetics, the solvent system also determines the amount of residual organic species that may remain trapped after film formation.^27^ Strongly coordinating solvents with high boiling points, such as DMF and DMSO, are known to form stable PbI_2_–solvent adducts that may not be completely removed during low-temperature processing.^27^ Residual solvent molecules have been associated with increased trap density, ionic migration, and reduced operational stability of perovskite solar cells. In contrast, the present solvent strategy combines two highly volatile solvents (2ME and ACN) with a limited amount of NMP. The rapid evaporation of ACN and 2ME is expected to reduce solvent retention,^22^ while the lower donor number of NMP compared with DMSO provides sufficient coordination to regulate crystallization without excessively stabilizing intermediate complexes.^26^ Although FTIR characterization was not performed in the present work, the improved crystallinity, lower optical scattering, and enhanced photovoltaic performance observed for the optimized 2ME:ACN:NMP composition are consistent with a more efficient solvent removal process reported for volatile solvent systems in previous studies, as can be seen in Table 4.

**Table 4:** Expected Residual Solvent Tendency

**Solvent System**	**Typical Scalable Deposition**	**Expected Residual Solvent Tendency**	**Main Evidence**	**Refs.**
DMF/DMSO	Spin Coating, Slot-Die	High	The high boiling point and strong coordination of DMSO favor the formation of PbI_2_-DMSO adducts that can remain after annealing.	^22,25,27^
2ME/DMSO	Blade Coating	Moderate	2ME accelerates evaporation, while DMSO maintains coordination during crystallization. The amount of DMSO should be optimized to avoid residues.	^22,25^
2ME/NMP	Blade Coating	Moderate-Low	NMP has a lower donor number than DMSO and forms less stable complexes, facilitating better PVK-ink stability.	^21,26^
*2ME/ACN/NMP* **(This Work)**	*Bar Coating*	*Expected Lower*	*The high volatility of ACN and 2ME favors rapid solvent extraction*, *while NMP controls crystallization*. *This trend requires experimental confirmation using FTIR.*	^22,27^

Despite the progress presented, there remains considerable opportunity to further optimize this approach, through advanced solvent engineering strategies, particularly by incorporating suitable additives to regulate crystallization, suppress degradation pathways, and enhance device performance.^46,47^ Such strategies could build upon the optimized processing parameters identified in the present work to further improve both the efficiency and operational stability. This provides a robust baseline for further efficiency enhancements through multication formulations or the incorporation of surfactants to further refine crystallization kinetics.^32,42,43,48^

To better position the present work within the current state of scalable perovskite manufacturing, Table 5 summarizes representative reports employing different large-area deposition techniques. Doctor-blade and slot-die coating currently achieve the highest efficiencies, primarily through sophisticated multication perovskite formulations, advanced additive engineering, controlled drying protocols, and optimized interface passivation. In contrast, the present work intentionally focuses on a simple MAPbI_3_ composition processed entirely glovebox-free, without crystallization additives or surfactants. Although the maximum PCE (11.85%) is lower than the current state of art, the proposed methodology demonstrates that careful optimization of solvent engineering together with bar-coating parameters can produce compact, homogeneous, and highly crystalline films using a considerably simpler processing route. Therefore, the present study provides a robust baseline that can be readily combined with modern compositional engineering and interface passivation strategies to further improve device performance while preserving process scalability.

**Table 5:** Benchmarking of Our Bar-Coated MAPbI_3_ Solar Cells against Representative Large-Area Perovskite Fabrication Techniques Reported in the Literature, Including Doctor-Blade, Slot-die Coating, Screen Printing, and Related Scalable Deposition Methods

**Technique**	**Perovskite**	**Atmosphere**	**Active Area**	**PCE (%)**	**Main Strategy**	**Year**	**Refs.**
Doctor Blade	FA/MAPbI/Br	Air		23.14	Additive MACl.	2023	^12^
Bar Coating	FA/MAPbI/Br	Air	Mini Module 15 cm^2^	20.99	Additives 1-cyclohexyl-2-pyrrolidone (CHP) and acetylcholine bromide (AcBr).	2021	^23^
Bar Coating	MAPbI_3_	Air	0.12 cm^2^	15.6	Single-crystal ink, hot air blower and a surface passivator (TiCl_4_) of TiO_2_.	2023	^13^
Slot-Die	MAPbI_3_	N_2_	0.16 cm^2^	20.83	2ME/DMSO ink	2021	^25^
Screen Printing	FAPbI/Br/Cl	Air	0.05 cm^2^	21.8	Weak solvent interaction with methylammonium acetate (MAAc).	2025	^9^
Inkjet	FA/MAPbI/Br	Air	0.126 cm^2^	13.44	Ambient printing with ternary solvent system (DMF/DMSO/ACN or 2ME) at 150 °C.	2025	^8^
*Bar Coating*	*MAPbI*_3_	*Air*	0.*104 cm* ^2^	11.9	*Additive-free*, *low-temperature*, *2ME/ACN/NMP.*	2026	***This Work***

## 4 Conclusions

This work presents a systematic solvent engineering strategy for the fabrication of MAPbI_3_ perovskite films by bar coating under ambient laboratory conditions without the use of a glovebox, humidity control, or antisolvent treatment. The results demonstrate that the crystallization behavior of the perovskite is governed by the synergistic interaction between solvent chemistry and the processing parameters of the bar-coating technique, highlighting the importance of optimizing these variables simultaneously rather than independently.

It is concluded that the combined use of 2-methoxyethanol (2ME), acetonitrile (ACN), and *N*-methyl-2-pyrrolidone (NMP) enables precise control of solvent evaporation and crystallization kinetics, while the substrate temperature and bar groove depth regulate film drying and crystal growth. Correlating structural, optical, morphological, and photovoltaic characterization allows the identification of the optimum processing conditions, consisting of a 2ME:ACN volume ratio of 70:30, 40 μL mL^–1^ of NMP, a substrate temperature of 30–40 °C, and a 12 μm groove depth bar. These conditions produce compact, highly crystalline, and optically homogeneous MAPbI_3_ films, yielding a maximum power conversion efficiency of 11.85% under moderate ambient relative humidity. Beyond identifying an optimized processing window, this work establishes practical relationships between solvent engineering, crystallization kinetics, and bar-coating parameters that provide a robust framework for ambient fabrication of perovskite thin films.

Although the present study intentionally excludes crystallization additives and compositional engineering to isolate the effects of the processing variables, the proposed methodology constitutes a reliable platform for future integration with additive engineering, interface passivation, and advanced perovskite compositions. Consequently, these findings contribute to the development of scalable and reproducible fabrication strategies for high-quality perovskite films and represent an important step toward the large-scale manufacturing of perovskite solar cells under ambient conditions.

## Supplementary Material



## Notes

Additional experimental details and complementary characterization results supporting the discussion in the main text are provided in the supporting information. XRD patterns, supporting information UV–Vis absorbance, transmittance, and reflectance spectra for all processing variables, and additional surface profile analyses (PDF)
