# Supplementary material for: Solvent and Bar Coating Parameter Selection for Optimum Deposition of Perovskite Films at Low Temperatures under Ambient Conditions
Source: ACS Omega. 2026 Sep 12;11(38):57604–16. doi: 10.1021/acsomega.6c06331 (PMC13629400; doi:10.1021/acsomega.6c06331)
Supplement: Supplementary file 1 [file ao6c06331_si_001.pdf]

Solvent and bar coating parameter selection for optimum deposition of perovskite films at low temperatures under ambient conditions

Carlos F. Arias-Ramos[1], Francisco E. Cancino-Gordillo[1], Diana Marcela Montoya[2], Paola G. Abrego-Martínez[1], Ari M. Vargas-Sotomayor[1], Asiel N. Corpus-Mendoza[1,3], Hailin Hu[1].*

[1*]Instituto de Energías Renovables, Universidad Nacional Autónoma de México. Priv. Xochicalco S/N, 62580, Temixco, Morelos, México.

[2]Grupo de Películas Delgadas y Nanofotónica, Departamento de Física, Pontificia Universidad Javeriana, Cr. 7 No. 40- 37, Ed. 52 Of. 606, Bogotá, Colombia.

[3]Secretaría de Ciencia, Humanidades, Tecnología e Innovación (SECIHTI), Av. Insurgentes Sur 1582 Col. Crédito Constructor, Demarcación Territorial, Benito Juárez, 03940, Ciudad de México, México.

* *Corresponding author:* cafaar@ier.unam.mx

The surface roughness (R_a) of the perovskite films[1] was estimated from the profiles obtained by profilometer, according to equation S1:

$$R_a = \frac{1}{L}\int_0^L |z(x) - m|\, dx, \qquad \text{(Eq. S1)}$$

where L is the sampling length of the profile, z(x) is the surface profile, and m is the centerline or mean line obtained from equation S2:

$$m = \frac{1}{L}\int_0^L z(x)\, dx. \qquad \text{(Eq. S2)}$$

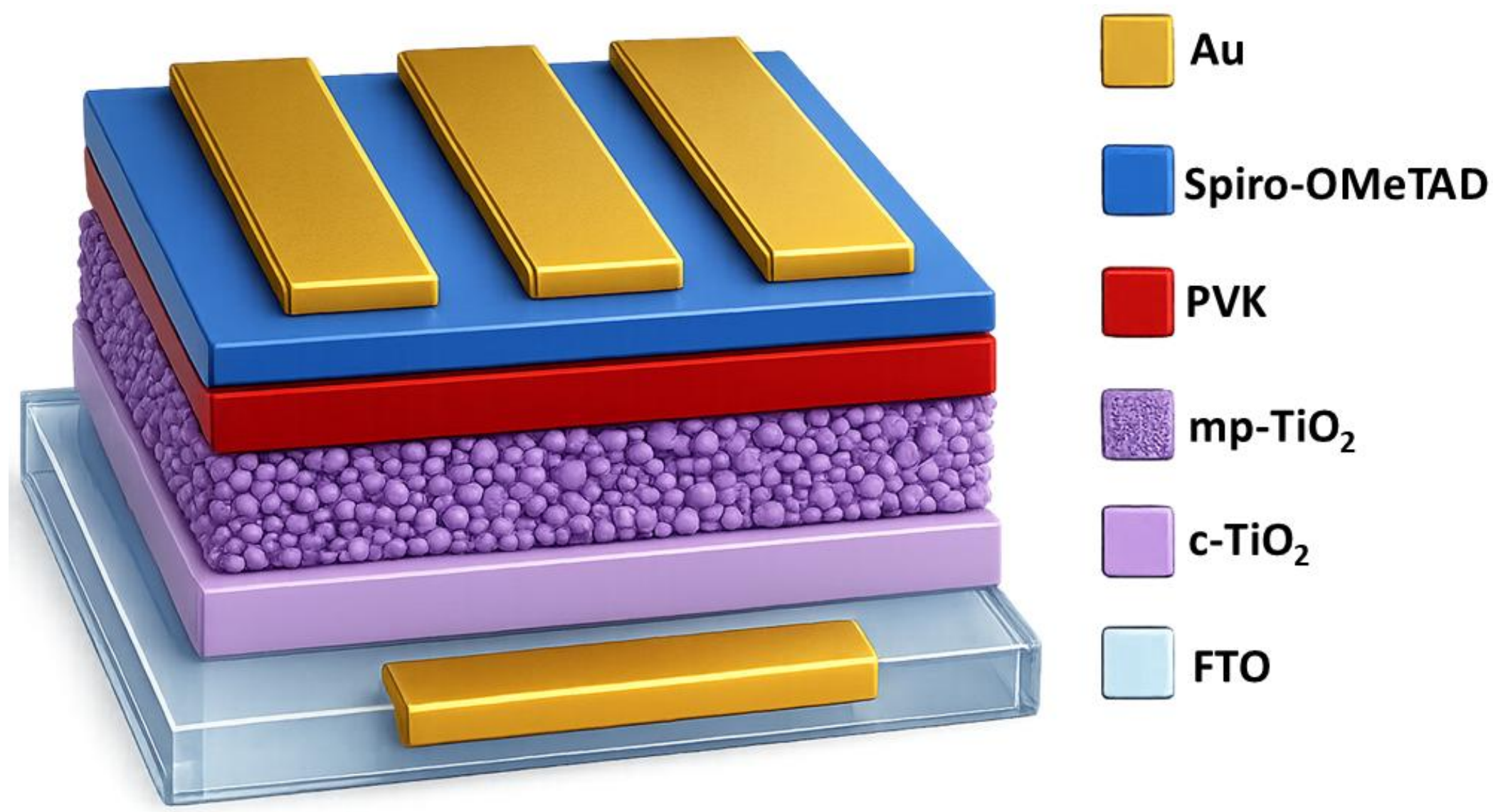

Figure S1. Scheme of the PSC architecture used in this work.

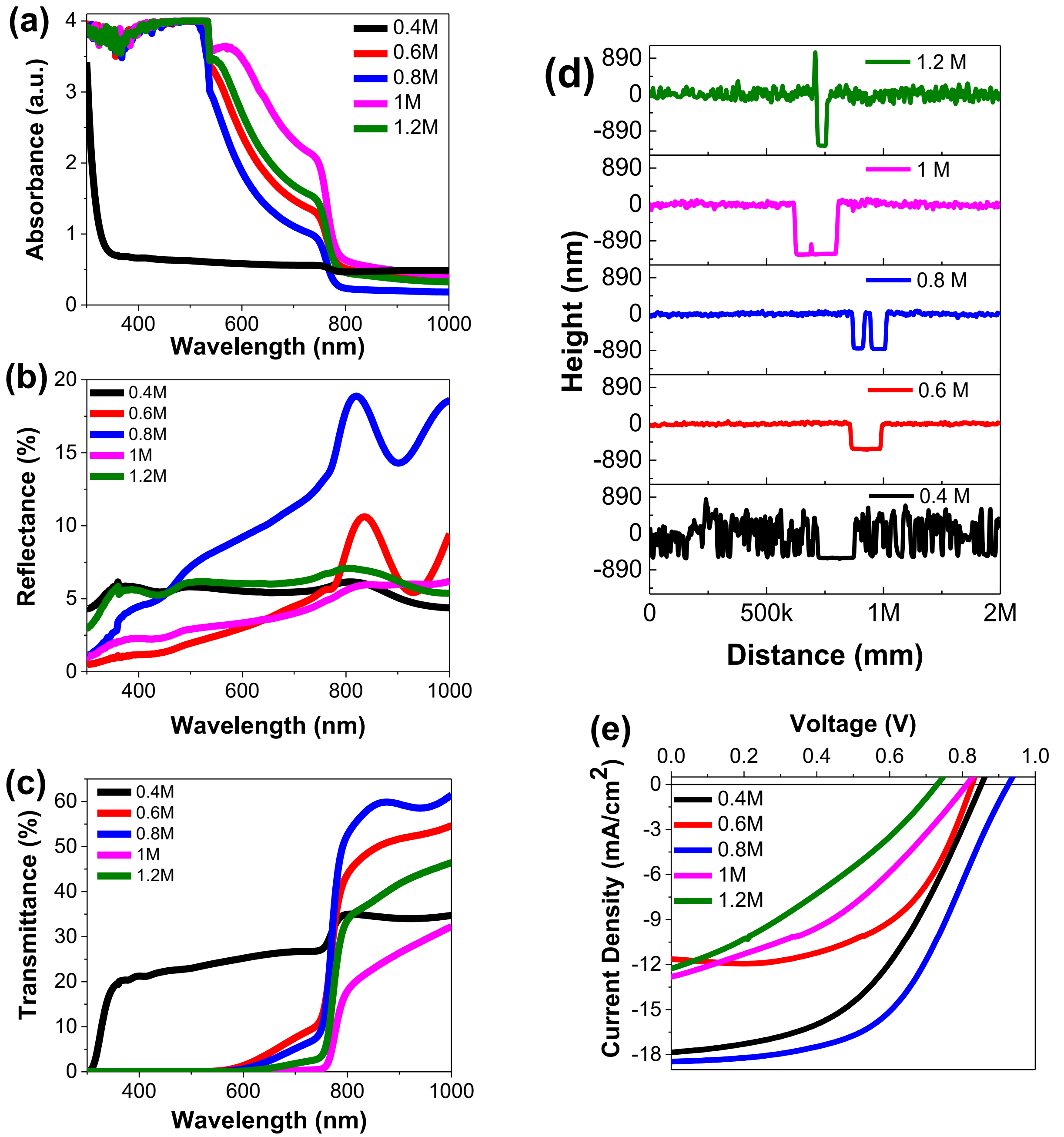

Figure S2. a) Absorbance, **b)** Reflectance, **c)** Transmittance, **d)** Film surface profile and **e)** J-V curves of solar cells prepared with different concentrations of the perovskite precursor solution (ink) (M = mg/mL). The solvent system was 2ME:ACN = 70:30, and NMP of 5 μL/mL. Deposition conditions: temperature = 25 °C, and relative humidity = 37%.

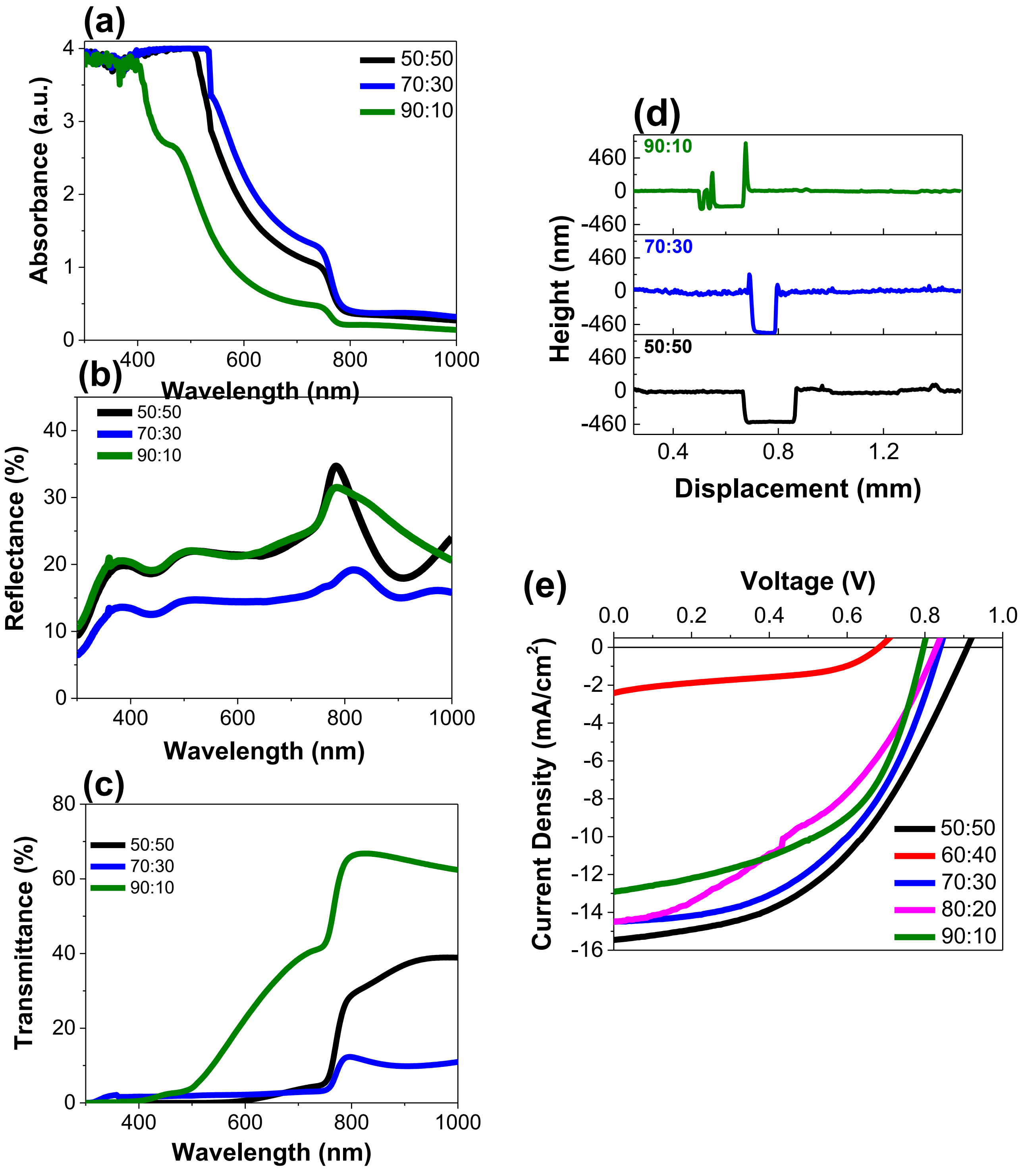

Figure S3. a) Absorbance, **b)** Reflectance, **c)** Transmittance, **d)** Film surface profile and **e)** J-V curves of solar cells prepared with different % v/v of 2ME:ACN = 50:50, 70:30 and 90:10. Deposition conditions: temperature = 23.9 °C, and relative humidity = 39%.

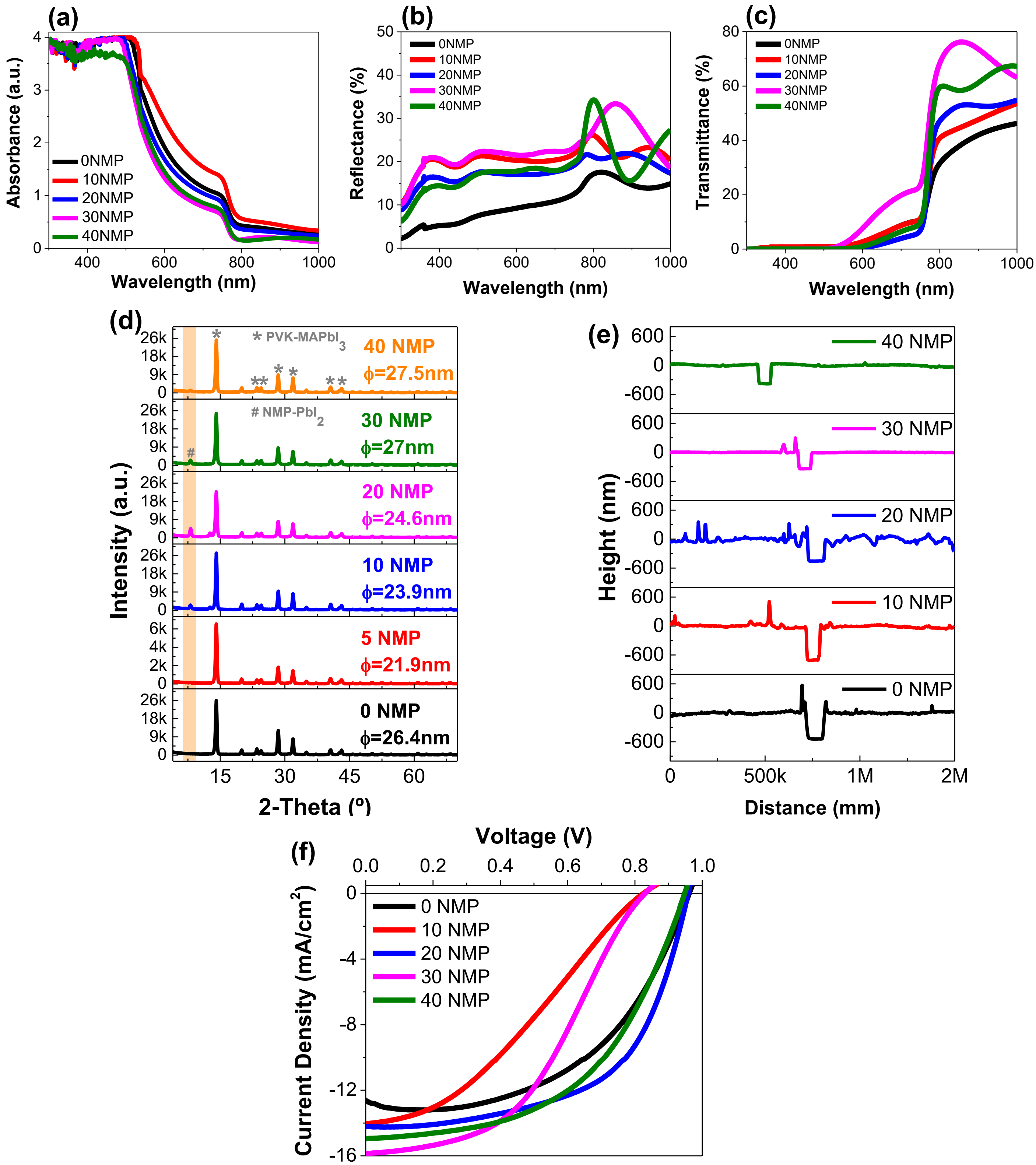

Figure S4. a) Absorbance, **b)** Reflectance, **c)** Transmittance, **d)** XRD patterns, **e)** Film surface profile and **f)** J-V curves of solar cells prepared with different concentration of NMP: 0 to 40 μL/mL. Deposition conditions: temperature = 23.8 °C, and relative humidity = 37%.

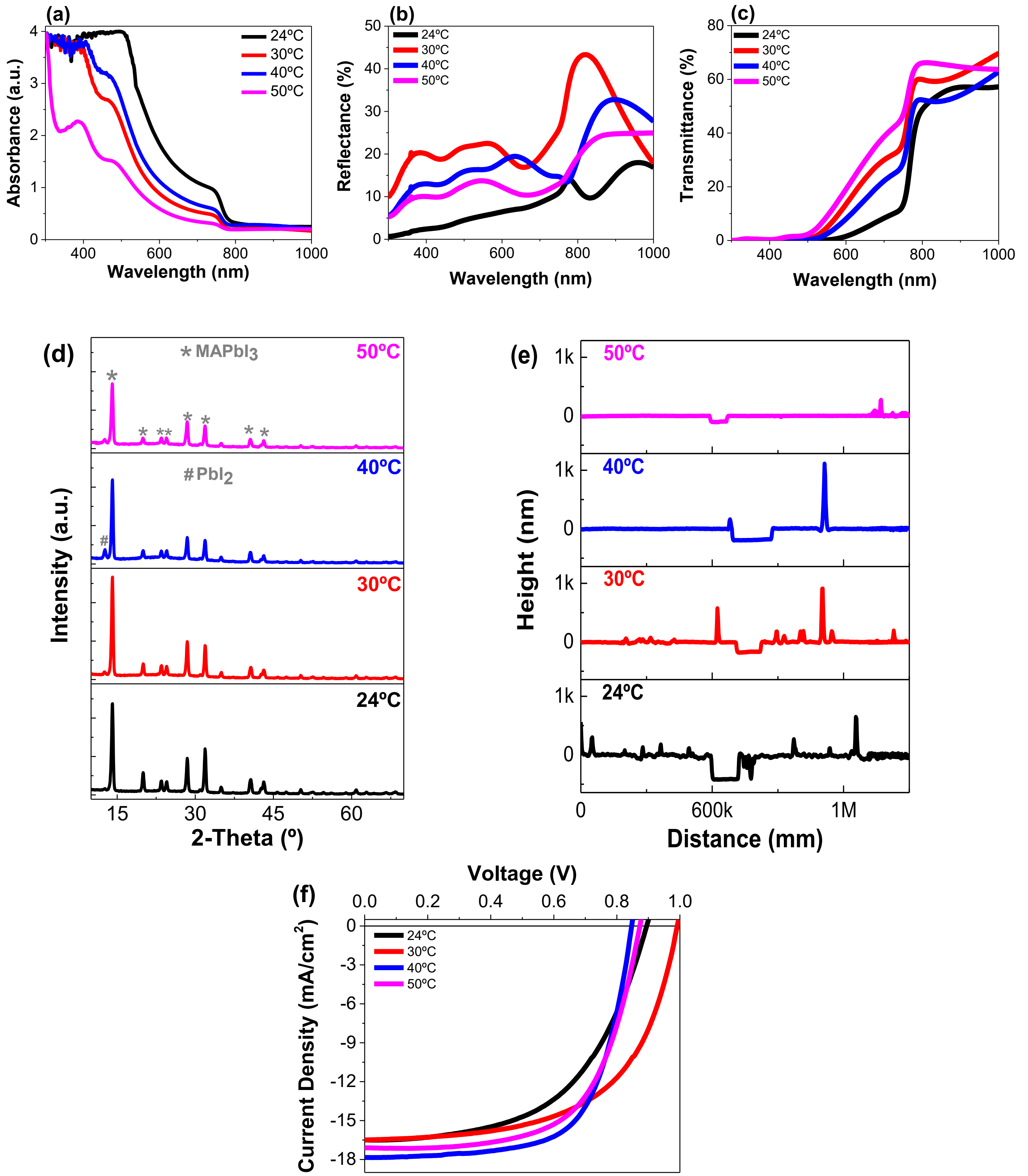

Figure S5. a) Absorbance, **b)** Reflectance, **c)** Transmittance, **d)** XRD patterns, **e)** Film surface profile and **f)** J-V curves of solar cells prepared at plate temperature of 24, 30, 40 and 50 °C. Deposition conditions: temperature = 23.4 °C, and relative humidity = 34 %.

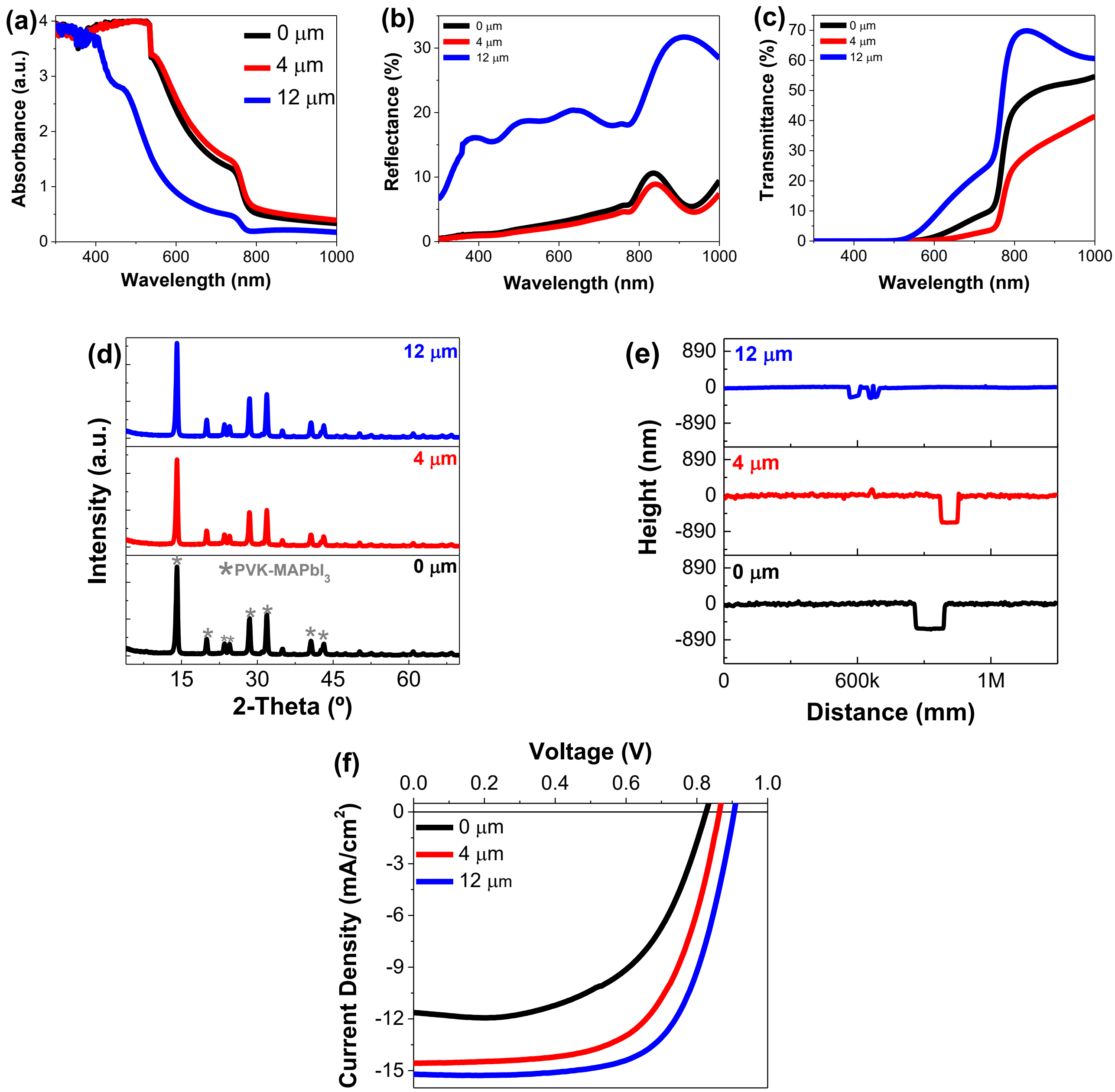

Figure S6. a) Absorbance, **b)** Reflectance, **c)** Transmittance, **d)** XRD patterns, **e)** Film surface profile and **f)** J-V curves of solar cells prepared with different the bar groove depths: 0, 4 and 12 μm. Deposition conditions: temperature = 23.8 °C, and relative humidity = 37%.

REFERENCES

(1) Bhushan, B. *Modern Tribology Handbook, Two Volume Set*, 0 ed.; CRC Press, **2000**. https://doi.org/10.1201/9780849377877.
